# Microbial Biofilms as Barriers to Chronic Wound Healing: Diagnostic Challenges and Therapeutic Advances

**DOI:** 10.3390/jcm14228121

**Published:** 2025-11-17

**Authors:** Yasir Almuhanna

**Affiliations:** Department of Clinical Laboratory Science, College of Applied Medical Sciences, Shaqra University, Shaqra 11961, Saudi Arabia; yalmuhanna@su.edu.sa

**Keywords:** biofilm, chronic, wound, healing, diagnostic, therapeutic, microbiome, resistance

## Abstract

Wound healing is a complex and multistage process that incorporates precise cellular and molecular coordination. The presence of biofilms in chronic wounds adversely affects the wound healing process, as it prolongs the closure of the wound, thus sustaining chronic inflammation. Current data suggest that biofilms are present in almost all chronic wounds, which leads to significant challenges in diagnosis and treatment. Traditional detection methods, such as cultures and light microscopy, often fail to detect biofilms; however, sophisticated molecular and imaging techniques are constrained by their expense and accessibility. Chronic wound management, therefore, has progressed from conventional antimicrobial application to integrated methodologies that incorporate biofilm debridement, antibiofilm dressing, negative pressure wound therapy, and innovative enzyme or nanoparticle interventions. This review highlights the clinical significance of biofilm presence as a barrier in chronic wound healing, assesses diagnostic and therapeutic innovations, and stresses the urgent need to improve patient outcomes.

## 1. Introduction

Wound healing is a four-stage process aimed at restoring the integrity of damaged tissue following injury. The four phases are arranged as hemostasis, inflammation, proliferation, and remodeling. Each phase is characterized by distinct cellular processes and molecular interactions that are essential for successful healing [1].

### 1.1. Wound Healing Stages

The initial reaction to tissue injury is hemostasis, which aims to stop significant blood loss and establish a temporary matrix for restorative cell types (e.g., fibroblasts) that infiltrate into the wound site in the later stages. Hemostasis commences with vasoconstriction and culminates in the formation of a fibrin clot. This clot functions as both a physical barrier to blood loss and the temporary matrix needed by the infiltrated cells to be involved in the wound healing process. Platelets are essential in this phase because they adhere to the endothelium and aggregate to create a plug. Upon activation, platelets release granules containing platelet-derived growth factor (PDGF) and transforming growth factor-β (TGF-β), which serve as immune cell recruitment signals and drive the subsequent stages of the wound healing process [1].

Hemostasis is followed by the process of inflammation, which is marked by the cellular infiltration of neutrophils and macrophages at the wound site. This phase is critical for preventing infection, and it further prepares the wound environment for healing. Arriving neutrophils start the process of phagocytosis of debris and bacteria. Neutrophils play a very important role as they produce reactive oxygen species, which are critical in bacterial killing. Additionally, they release effector cytokines such as interleukin (IL)-1β, TNF-α, and IL-6, which amplify inflammation and recruit additional immune cells [2]. Subsequently, macrophages arrive to orchestrate the healing process, as these cells engulf and remove apoptotic cells and debris. The macrophages also release regulatory cytokines and growth factors, such as IL-1β, which promote further cell migration and affect fibroblast activation and differentiation, but their tissue-specific effects are unclear [3].

Following inflammation is the proliferative phase of wound healing, which is characterized by the formation of new blood vessels (angiogenesis), collagen deposition, granulation tissue formation, and epithelization. Fibroblasts are needed in this stage as these cells deposit a new extracellular matrix (ECM), which replaces the transient platelet plug established during hemostasis. Resident endothelial cells proliferate for angiogenesis, which is stimulated by growth factors, including vascular endothelial growth factor (VEGF) and fibroblast growth factor (FGF). The formation of new blood vessels is crucial for supplying nutrients and oxygen to the new tissue. Generating an ECM and new cells is very energy-intensive; therefore, a new supply of oxygen and nutrients is needed to support granulation and restoration of the wounded tissue. Simultaneously, keratinocytes migrate across the wounded bed to restore the epidermal barrier, a process aided by keratinocyte growth factor (KGF) and epidermal growth factor (EGF). The formation of a new epidermal barrier is essential as it inhibits fluid loss from the wound, which, if not stopped, can further damage the wounded tissue and serve as a breeding ground for pathogenic bacteria [4,5].

The last step of the wound healing process is remodeling, in which the newly formed tissue is refined and strengthened. In this stage, collagen, a major structural component of the ECM synthesized by fibroblasts, is reorganized and crosslinked in order to enhance the stretchability of the wound surface. The ECM works as the structural framework that facilitates cell motility, adhesion, and signaling during healing, and a proper balance between ECM deposition and degradation is essential to restoring tissue integrity. Matrix metalloproteinases (MMPs) catalyze the proteolytic degradation of ECM proteins, while their inhibitors, the tissue inhibitors of metalloproteinases (TIMPs), regulate this process. This dynamic balance between MMPs and TIMPs allows the ECM to be reshaped into mature scar tissue, and the remodeling process may persist for months to years following the initial injury, depending on the extent of the wound and the healing response [6,7].

### 1.2. Factors Delaying Wound Healing

**Figure 1:**
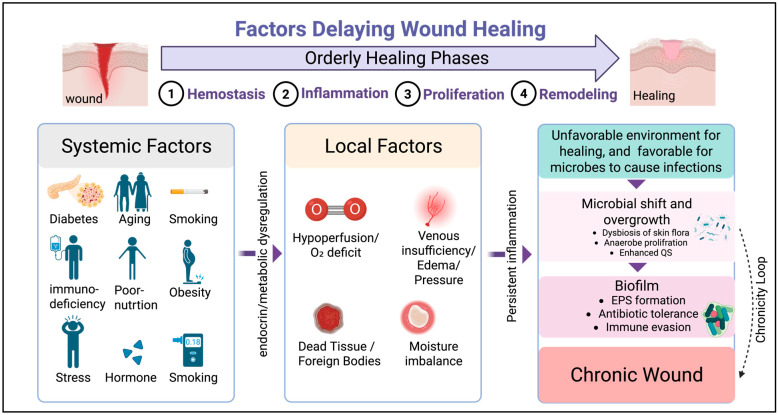
Factors delaying wound healing. A wound that fails to progress sequentially through the phases of healing is considered chronic [8]. Systemic risks include diabetes, age, hormones, stress, obesity, smoking, alcohol, malnutrition, and immunocompromised status which alter local wound microenvironment. Local factors include poor perfusion/O_2_ deficiency, venous insufficiency, and dead tissue. All these factors turn the wound environment in favor of persistent infection, promote microbial dysbiosis and initiation of biofilm. This establishes a cycle of delayed healing [9,10]. (Created with BioRender).

Any wound that fails to progress through the four stages of healing in their sequential manner is considered a chronic wound [8]. Several factors affect whether a wound progresses from inflammation to proliferation and remodeling, thus becoming a chronic wound. These factors are related and are grouped into two categories, which are systemic factors (host level) and local factors (wound bed) [7,9]. Collectively, these factors reshape the wound microenvironment in a way that supports infection. Systemic factors are related to the overall health or status of the individual, such as age, diabetes, hormones, stress, obesity, medication, smoking, alcohol, immunocompromised status, and nutrition [9,10]. Systemic factors maintain the inflammation status, raise oxidative stress, and reduce the body’s ability to make new vessels and collagen [9,10]. This results in reduced effectiveness of white blood cells, weakened growth factor signaling, and hampered capacity of the wound to progress to the healing phases [9,10]. Local factors, meanwhile, are those related to the wound environment itself, such as impaired oxygenation, venous insufficiency, foreign bodies, and infection. Recent evidence indicates that chronic wound persistence involves cellular senescence, oxidative stress, and impaired angiogenesis forming a self-sustaining inflammatory cycle [11]. Furthermore, matrix mechanics and disrupted mechano-transduction within the extracellular matrix microenvironment have been shown to perpetuate chronicity and fibroblast dysfunction [12]. Together, these systematic and local issues put the wound in the inflammation phase as oxygen and nutrients are limited, cell migration is hindered, and the chemical environment (pH, enzymes, reactive oxygen species, etc.) turns unfavorable for healing, becoming a favorable environment for microbes to cause infections, one of which is the focus of this review—biofilms (Figure 1) [9,10]. Unlike previous work that separately discusses microbiological or clinical aspects of chronic wounds, the present review provides an integrated framework linking microbial biofilm mechanisms with diagnostic and therapeutic innovation. Thus, the aim is to draw attention to translational aspects where improved standardization and scalability could enhance clinical application.

## 2. Biofilms in the Context of Chronic Wounds

Research indicates that 60% to >90% of chronic wounds contain biofilms [13,14]—organized microbial cells, frequently of multiple species, that attach to the wound surface. Biofilms are believed to be responsible for hindering the healing of all chronic wounds and interfering with normal tissue repair mechanisms [15,16]. In addition to delaying the healing process, biofilms sustain a detrimental wound microenvironment through molecular, cellular, and structural disturbances. These complex, structured communities are embedded within a self-produced extracellular matrix consisting of an extracellular polymeric substance (EPS) that includes proteins, DNA, and polysaccharides. The EPS has significant effects on the wound healing process, as it provides resistance against the immune defenses of the wounded host and against conventional antimicrobial agents. The protection provided by this environment allows the biofilm microorganisms to thrive and resist treatments that would typically clear the wound of planktonic, free-living bacteria. Moreover, the EPS plays an important part in maintaining a chronic infection state and hinders the normal stages of healing [16,17]. The resilience of biofilms is likely due to the ability of the EPS to slow down the penetration of antimicrobial agents alongside mutations in the genetic and metabolic state of the embedded bacteria, which often become dormant and thus less susceptible to treatments that target planktonic bacteria [18,19]. The aims of this review were to highlight the clinical significance of biofilms in chronic wounds, evaluate recent diagnostic and therapeutic advancements, and emphasize the urgent need to overcome adverse healthcare outcomes attributable to biofilm formation in wounds.

## 3. Understanding Biofilms

The biofilms produced by different bacterial species display various shared features, including EPS generation, structural organization, metabolic and genetic mutations, and adaptations [20]. Characterization of biofilms formed by different bacterial species, such as *Escherichia coli*, *Pseudomonas aeruginosa*, and *Staphylococcus aureus*, suggests that these microorganisms utilize shared methods for biofilm formation [21,22]. The different substances in the EPS matrix (e.g., proteins, polysaccharides, and extracellular DNA) have different functions, ranging from providing structural integrity and mediating surface adhesion to working as a protective barrier against threats to bacterial survival (e.g., antimicrobial agents and the host immune system). Studies have shown that variations in EPS composition between species can impact the physical properties and protective capabilities of the resulting biofilms. For example, alginate, an EPS component of *P. aeruginosa* biofilm, enhances viscosity and antibiotic tolerance, whereas poly-N-acetylglucosamine (PANG), which is a major biofilm component of *S. aureus*, promotes bacterial immune evasion [23]. Similarly, crystalline EPS layers formed by Proteus mirabilis on the surface of catheters and the host promote mineral deposition and delay healing [24]. Biofilms exhibit highly organized architectures that contribute fundamentally to the efficient functioning of biofilm and regulate nutrient acquisition and waste disposal. Imaging analysis has revealed a constant cellular arrangement across different bacterial species, suggesting shared mechanical principles that regulate biofilm structures [21].

Bacteria growing within biofilms express unique metabolic properties that are different from those of their planktonic (free-living) counterparts. For example, biofilms show improved efficiency in nutrient utilization, a very important metabolic adaptation that is crucial for biofilm resilience and survival in stressful environments, such as those with limited nutritional availability. It is important to mention that the wound area is considered a highly stressful environment, marked by persistent immunological reactions, variable oxygen levels, and limited nutrients. Microorganisms in biofilms show increased consumption of glucose while concurrently diminishing the use of amino acids. These changes in metabolic processes are facilitated by modifications in the microbial gene expression profiles, which particularly adapt it to the biofilm mode of growth; these changes in genetic profiles may vary significantly across various bacterial species [25].

All these features are orchestrated through intercellular communication within the biofilm and are mediated through quorum sensing, which is a kind of intercellular communication among bacteria. This communication capability enables the bacteria within the biofilm to coordinate their behavior and enhance their responses to surrounding changes and is essential for the development, maintenance, and function of biofilms. The detailed regulatory pathways involved in this communication can differ significantly among different bacterial species and might be affected by microbial adaptation to different niches [21].

## 4. Stages of Biofilm Formation on Wounds and Commonly Involved Microbial Species

Experimental studies have reported that biofilms build up on the surfaces of chronic wounds through a dynamic process that might incorporate several stages and contribute to the complexity and resilience of the biofilm structure. However, it is not yet clear whether the process reported in laboratory-controlled settings and animal models is the same process followed by the organism in clinical settings [15].

The reported procedure starts with the initial microbial attachment, followed by microcolony development and matrix production, and eventually the dispersion of single cells [15]. It has been reported that *P. aeruginosa* attaches to the wound surface within hours in laboratory settings, with the EPS detected as early as five hours after attachment [26]. The EPS works as a protective matrix that envelops bacterial communities and augments their resilience to antimicrobial agents and the host immune response, as stated earlier. During the early stage, some Gram+ and Gram− bacteria might be observed; however, as the biofilm fully matures, it becomes increasingly overtaken and dominated by *P. aeruginosa* and *S. aureus* [18,27,28].

Mature biofilm can take up to 3 days [26,29]. However, the timeline differs across models; in in vitro studies, a fully mature biofilm has been observed over 1 h to 2 days, while in animal studies, it has been reported that significant microcolony formation occurs within a period of 3 to 7 days [30,31]. In the advanced stage, robust microcolonies that are firmly embedded within a compact EPS have been observed. In vitro studies indicate that this phase could take between 10 and 20 h, whereas the same process may extend from 7 to 20 days in vivo [32]. During this phase, *P. aeruginosa* and *S. aureus* remain the primary species, while several other anaerobes might be observed [31].

Few studies have investigated the microbial composition within wounds from patient samples. In all studies, the most dominant strains were *S. aureus* and *P. aeruginosa*, respectively, with other strains also being reported. Recent investigations have demonstrated that chronic wound biofilms are polymicrobial. Fluorescence in situ hybridization (FISH) and metagenomic profiling studies have revealed that *P. aeruginosa* and *S. aureus* frequently coexist with strict anaerobes such as *Finegoldia magna*, *Peptoniphilus*, and *Anaerococcus* spp., as well as skin commensals including *Corynebacterium* spp. and coagulase-negative *Staphylococcus* species, forming highly structured multispecies communities [27,33]. These microorganisms engage in metabolic cross-feeding and redox balancing, which creates micro gradients of oxygen and pH that promote survival of anaerobic partners in deeper wound layers [34]. Spatial imaging confirms niche partitioning with *S. aureus* dominating the superficial oxygen-rich biofilm surface, whereas *P. aeruginosa* and anaerobes colonize hypoxic or necrotic tissue zones, maintaining chronicity [19]. Such interspecies cooperation was reported to enhances biofilm density and enable the biofilm to grow faster [35]. The observed microbial diversity within the chronic wound may be attributed to the resident normal flora, as it has been reported that different microbial normal flora occupies the skin [29]. However, the differences in analytical techniques and sampling methods across these studies may affect the reported compositions of microbial communities.

A deeper understanding of the different stages of biofilm development in chronic wounds and the microbial species involved at each stage could provide essential insights that might aid in chronic wound treatment. Any future management approaches must account for the constantly changing nature of microbial communities during biofilm development within the wound. In particular, examining the different stages of biofilm formation, particularly the initial attachment and colonization processes, could yield novel therapeutic intervention approaches that could prevent the establishment of robust biofilm communities, therefore improving the clinical results for individuals struggling with chronic wounds.

## 5. How Biofilms Hinder the Natural Process of Wound Healing

This section brings together findings from clinical, in vivo, and in vitro studies to clarify how biofilms exert these effects. Biofilms block crucial healing processes and modulate inflammatory signaling. In vivo studies have shown that biofilm formation increases the levels of pro-inflammatory cytokines such as IL-1β, TNF-α, and IL-6 while inhibiting important immunomodulators, including TLR-2, TLR-4, S100A8, and S100A9 [36,37]. These alterations prevent the necessary shift toward wound healing by prolonging the inflammation status.

Additionally, biofilms block cell migration, which is critical for tissue regeneration [38]. It has been reported that the movement of keratinocytes and fibroblasts is notably diminished in the presence of biofilms or products derived from biofilms [30,39]. This eventually leads to a delay in re-epithelialization and the formation of granulation tissue. In vitro models further support this finding, showing that biofilm-conditioned media inhibit keratinocyte proliferation and migration. Another significant interference mechanism is the degradation of the ECM. Biofilms have been reported to enhance the expression of ECM metalloproteinases while reducing collagen synthesis through microRNA pathways, resulting in compromised ECM integrity and increased wound fragility [40,41].

Neutrophils are among the first cells recruited to the wound site during the inflammation process, where they play a very important role in bacterial clearance through phagocytosis, the release of effector cytokines, and the generation of reactive oxygen species. However, the presence of a biofilm restrains the function of neutrophils. It has been reported that biofilms can block the release of neutrophils, effector cytokines, and ROS, resulting in inefficient bacterial clearance and thus promoting persistent infection [42]. Nguyen [43] showed that this effect is amplified in diabetic models and contributes to the persistent infection associated with chronic diabetic wounds. Cytokine levels are also modulated, with dysregulation of both the pro-inflammatory cytokine IL-6 and the anti-inflammatory cytokine IL-10 in chronic wounds that contain biofilms [44]. This dysregulation is significant, as it results in a state of chronic unresolved inflammation that hinders the transition to tissue repair.

**Figure 2:**
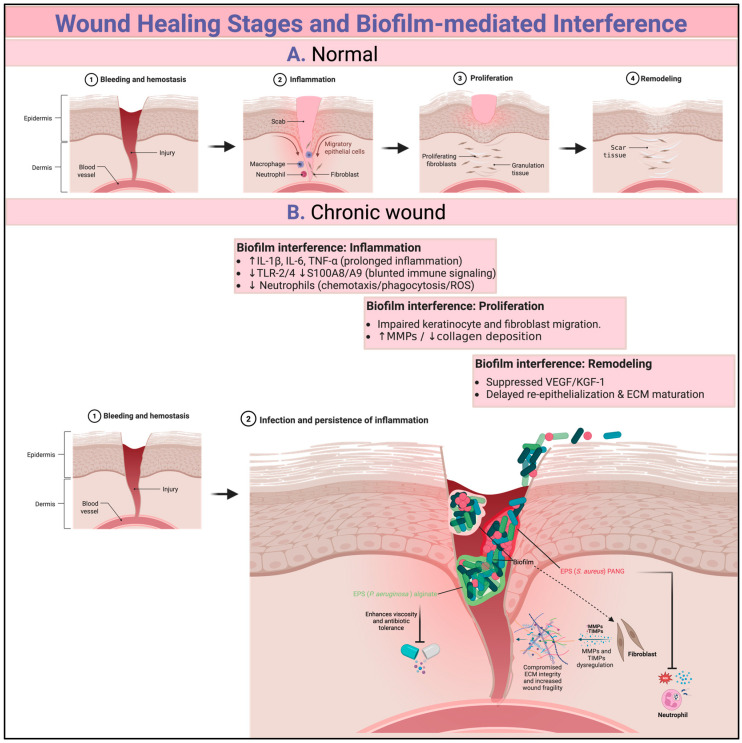
Wound healing stages ((**A**). Normal) and biofilm-mediated interference ((**B**). chronic wound). Biofilms prolong inflammation and blunt innate signaling, while impairing neutrophil functioning and keratinocyte/fibroblast migration and increasing ECM degradation, which collectively hinder and delay re-epithelialization and remodeling. (Created with BioRender).

As stated previously, the EPS surrounds the biofilm and acts as a barrier against immune cells and antimicrobial agents. This protective role of the EPS was highlighted and reported that disrupting the EPS improves antimicrobial efficacy and wound healing in an in vivo rabbit ear model [26,45]. Biofilms can also release toxins and enzymes that suppress or degrade crucial signaling and structural proteins, including growth factors such as VEGF and KGF-1 [46]. This interference results in necrosis-impaired angiogenesis and further delays tissue regeneration. Finally, changes in the surrounding wound bed also contribute to impaired healing. Biofilm-infected wounds are characterized by hypoxia, oxidative stress, and pH imbalances, which suppress fibroblast activity, promote necrosis, and further hinder the wound healing process [27]. Figure 2 summarizes the key interference mechanisms that contribute to delayed wound healing. Recent multi-omics studies have obtained data that define both microbial and host determinants of chronic wound biofilm. In particular, data from a chronic wound characterization study combining transcriptomic and proteomic analyses revealed persistent upregulation of innate immune and ECM remodeling pathways, along with high levels of neutrophil-derived proteases and complement proteins, findings consistent with a self-sustaining inflammatory state [47]. Complementary proteomics profiling of wound slough demonstrated high levels of MMP, neutrophil elastase and bacterial proteins associated with biofilm formation, indicating that slough represents a biofilm-rich niche rather than tissue debris [48]. Collectively, these findings highlight conserved microbial and host pathways that sustain chronicity.

## 6. Barriers to the Diagnosis of Biofilms in Chronic Wounds

The effective detection of biofilms remains one of the most significant challenges to successful clinical care of chronic wounds. Although the presence of biofilms has become increasingly recognized as an important inhibitor of healing, both practical and technical limitations hinder their accurate identification and characterization. This section compiles key findings that bring together important insights from recent research while emphasizing methodological shortcomings, undesirable variation in detection rates, and the clinical implications of diagnostic ambiguity.

Traditional diagnostic approaches, including standard bacterial cultures and basic microscopy, have demonstrated consistent weaknesses in detecting biofilms. Several studies have reported that lab bacterial cultures often fail to replicate the diversity of microbial communities within biofilms clinically, mainly due to difficulties associated with yielding the fastidious or slow-growing organisms from the sample [18,49]. In addition, Gram staining and basic microscopy lack the resolution and structural clarity needed to differentiate multiple bacterial cells embedded in the EPS to form biofilms from planktonic single cells. These constraints often result in misinterpretation of the sample and false negatives. In addition, the location of the biofilms within the wound intensifies the detection challenge in terms of sample collection. For example, superficial swabbing may not capture deeply embedded or patchy biofilm colonies, which are easily missed by surface sampling methods [19,33]. Consequently, opportunities for timely intervention may be missed, leading to prolongation of the inflammation and chronic status.

Conversely, modern molecular and imaging techniques, although providing improved detection precision, are often inaccessible for regular use. Many methods, including peptide nucleic acid fluorescence in situ hybridization, confocal laser scanning microscopy, and next-generation sequencing, have shown exceptional effectiveness in identifying species composition and biofilm structure. For instance, these technologies have been used successfully to identify polymicrobial biofilms, including those formed by strict anaerobes, in cases in which cultures fail [50,51]. Fluorescence imaging is gaining popularity as an imaging technique that shows promise in clinical settings. Recently, it was reported that identification of biofilms with fluorescence imaging has a sensitivity of 84% and 63% accuracy in identifying biofilms, vastly surpassing identification using clinical signs and various bedside techniques [52]. However, the broad adoption of these technologies is still restricted due to their high costs, the complexity of technology, and the requirement of specialized training and equipment [53].

In the absence of a valid, rapid, and widely accessible diagnostic tool, the chronicity of the wound itself has become the most practical and widely accepted clinical reference for the presence of biofilms. As stated earlier, chronic wounds are strongly associated with the presence of biofilms, which makes chronicity a useful marker in daily practice. Biofilms are detected in approximately ~80% of chronic wounds, which indicates a clear link between chronicity and biofilm presence [44]. It has been reported that all non-healing wounds harbor biofilms, reinforcing the use of chronicity as a diagnostic method [19,27,54].

**Figure 3:**
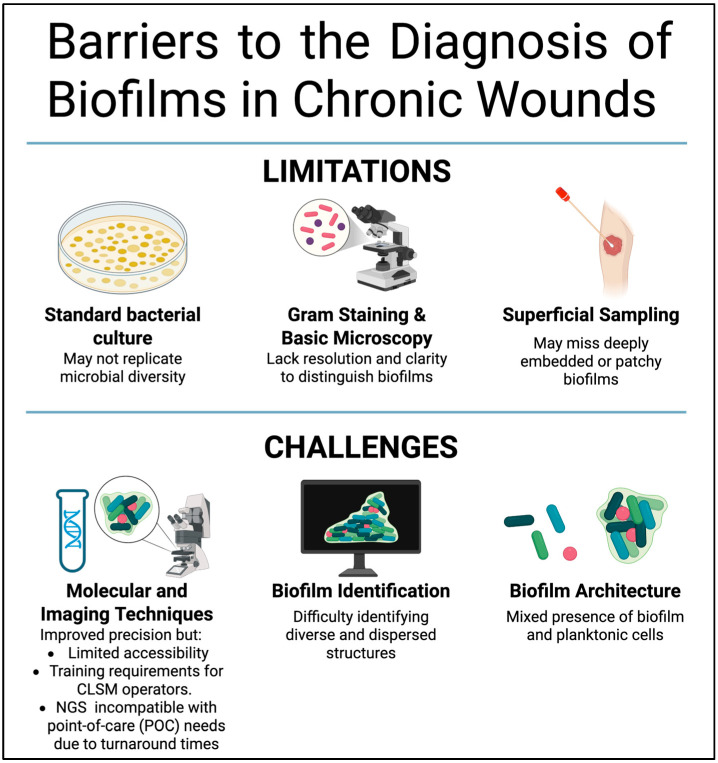
Barriers to the diagnosis of biofilms in chronic wounds. Overview of the conventional and emerging diagnostic methods for biofilms in chronic wounds and their challenges. Traditional methods, such as cultures and basic microscopy, provide limited sensitivity and fail to distinguish biofilms. Meanwhile, advanced molecular and imaging-based technologies enable more accurate and rapid detection; however, they have constraints such as cost, accessibility, and standardization issues. (Created with BioRender).

The identification of biofilm community composition and the architectural organization of biofilms from well-established matrix-enclosed colonies to scattered planktonic single cells also remains a significant challenge. Notably, the biofilm architecture, which significantly influences the visibility and recoverability of microbial communities, is further complicated by the coexistence of distinct bacterial clusters and single cells within the same wound [19]. This complexity underscores the urgent need for comprehensive methods that can go beyond binary detection to analyze the organization and viability of bacterial biofilm populations. These are features that traditional tools, with their inherent limitations, are unable to provide (Figure 3).

## 7. Current Treatment Strategies

To achieve a successful wound healing result for chronic wounds, it is necessary to follow a comprehensive approach that considers the reduction of the microbial burden and the prevention of biofilm reformation. One of the key approaches in biofilm treatment is the combination of both antimicrobial agents and anti-biofilm agents. Recent real world evidence from the Wound Hygiene Protocol Demonstrated that combining cleansing, debridement, and Aquacel Ag^+^ dressings diminished suspected biofilm coverage from 79% to 18% and provided an 80% reduction in wound volume after 31 days of treatment [55].

Mechanical debridement remains a fundamental step of managing biofilms in chronic wounds as it effectively disrupts the EPS, which is one of the major components of biofilm structure that plays a fundamental role in biofilm protection. It has been reported that the use of sharp monofilament fibers and ultrasonic debridement has considerable efficacy in reducing biofilm biomass. A reduction in biofilm burden after serial sharp debridement was reported [56], whereas it was observed that the post-debridement application of AQUACEL Ag^+^ resulted in a wound closure rate of 72.6% [57]. Similarly, Mori [58] demonstrated that a synergetic approach involving wound blotting and ultrasonic debridement markedly exceeds the healing efficacy of standard care.

The application of integrated methodologies, especially negative pressure wound therapy (NPWT), has demonstrated efficacy against intricate or stubborn wounds. A 47% reduction in biofilms following one week of NPWT with sodium hypochlorite instillation was documented [59], whereas a reduction in both the bacterial load and wound area when employing NWPT in conjunction with biofilm-disrupting agents was noted [60]. The significance of multimodal interventions were emphasized by documenting complete wound closure and a 76% graft retention rate after surgical debridement, skin grafting, and NPWT [61]. In parallel, new innovative diagnostic tools are improving clinical decisions and treatment plans. For example, fluorescence imaging has been reported to influence management strategies in 528 wound cases, representing 53.3% of all cases, resulting in better identification of bacterial hotspots [62]. Table 1 summarizes the biofilm-targeted therapeutics options for chronic wounds with reported outcomes, key cautions, and points of integration. Although variability in treatment duration and diagnostic criteria is already summarized in Table 1, these finding underscore the need for a harmonized evaluation framework that links biological efficacy, clinical outcomes, and scalability enable cross-study comparability and practical translation.

It has been reported that the use of antiseptics such as iodine and PHMB leads to positive results [68,69], while silver-based solutions have considerable anti-biofilm effects but may cause localized irritation [70]. Combination therapies enhance effectiveness, as observed in several studies [59,71,72]. Recent studies suggest the promising potential of novel treatment dialkylcarbamoyl chloride (DACC)-coated wound dressing [73] and p-toluenesylfonic acid [74]; however, further analysis is needed. Antibiotics frequently exhibit limited efficacy in penetrating biofilms, with systematic reviews indicating that they seldom exceed the performance of antiseptics [75,76]. However, when antibiotics are combined with antibiofilm agents, they result in significantly improved chronic wound healing [55].

Despite this advancement, challenges in the utilization of protocols still exist. The frequency and length of the current protocols show significant diversity, ranging from 2 to 12 weeks. Moreover, patient-specific factors, including wound chronicity, predisposing conditions, and individual tolerance to treatment, play a decisive role in the choice of therapy applied. Additionally, the lack of a standardized biofilm diagnosis protocol presents major challenges in selecting appropriate therapies and hampers the comparison of results across clinical studies.

It has been proposed that the best approach to handle chronic wound biofilm is the “step-down approach,” which is a multistage strategy that starts with repeated debridement aiming to disrupt microbial aggregates, followed immediately by the application of antimicrobial and antibiofilm agents to suppress the reformation of the biofilm [15]. Overall, the identified challenges highlight the pressing necessity for cohesive diagnostic criteria and uniform outcome measures to enhance strategies aimed at addressing biofilm-related wound care.

## 8. Potential Innovative Approaches for Managing Wound Biofilms

The effectiveness of enzyme-based treatments against biofilms has been highlighted by several studies. For example, glycoside hydrolases (PelAh and PslGh), which target the EPS, have been shown to reduce the biofilm biomass of *P. aeruginosa* by up to 58% to 94% and boost neutrophil killing by ~50%. Furthermore, these enzymes enhance the susceptibility of biofilms to colistin [65]. Another example is dispersin B, which, when combined with sodium dodecyl sulfate (SDS), shows efficacies of 50% to nearly 100% in reducing the biofilm biomass of several bacterial strains, including *S. aureus* and *E. coli* [66]. This synergistic effect demonstrates the possibility of a promising treatment approach that may be less susceptible to the development of biofilm resistance.

Other studies underscore the importance of using enzyme cocktails that combine more than one enzyme, each targeting different structural components of biofilms or causing a different effect on biofilm stability. It was reported that combination of matrix degrading enzymes including oxidoreductases, proteases, and polysaccharide hydrolases, produced synergetic effect in disrupting *S. aureus* and *P. aeruginosa* biofilms [77]. It was also indicated that enzyme cocktails targeting multiple biofilm components simultaneously enhanced both biofilm dispersal and bactericidal activity compared with single enzyme application [78]. This approach of combining more than one product provides disruption of the biofilm structure, which might enhance antibiotic penetration, thus offering another promising technique for tackling chronic wound infections. The noncytotoxic effect of these enzymes, as highlighted in previous research, suggests the potential for their use in clinical settings [79].

Another promising addition to the wound care arsenal is advanced wound dressings that contain a combination of silver with chelators, e.g., EDTA surfactants, benzethonium chloride, and pH regulators. This mixture of components works collectively to inhibit biofilm formation and has been reported to show higher performance when compared to conventional dressings. For example, this synergy significantly reduces the biofilm biomass of *S. aureus* and Klebsiella pneumoniae [64]. Likewise, iodine-based dressings can completely disrupt biofilms; however, their long-term efficacy depends on maintaining a controlled and sustained release that preserves antimicrobial potency while minimizing cytotoxic effect on the surrounding tissue [63]. Antibiofilm gels also provide a significant bacterial count reduction and are notable for their ability to maintain long-term biofilm disruption [80]. Another potential innovative approach for preventing biofilm formation is an electroceutical dressing, which may also modify bacterial gene expression. These dressings, which utilize a combination of silver, zinc, and a weak electric field, have shown encouraging results in animal models, highlighting their potential for tackling biofilms in clinical settings [67].

Various policies and guidelines emphasize the importance of timely and aggressive debridement to disrupt mature biofilms as a key aspect of managing wound biofilms [15,81]. Debridement is immediately followed by the application of topical antimicrobial agents to combat residual biofilm constituents [15]. It was demonstrated that structured biofilm-based wound care, which takes into consideration biofilm debridement and antibiofilm agents in the course of treatment, significantly improves healing rates [27]. This finding highlights the importance of considering biofilms as the major barrier to chronic wound healing and the need for biofilm-targeted management strategies.

The importance of the application of topical antimicrobials following debridement, such as silver, polyhexamethylene biguanide, iodine, and gentamicin, has been consistently recommended in several consensus guidelines. While debridement is essential, it is not sufficient on its own. For example, the use of topical antiseptics immediately after debridement while applying systemic antibiotics to tackle planktonic or systemic infection has been emphasized [82]. Furthermore, incorporation of additional strategies after debridement, such as NPWT and targeted antibiofilm agents, plays a crucial role in comprehensive wound management, as reflected in expert opinions [17]. These approaches address the biofilm directly and simultaneously aid in creating an environment that facilitates the healing process by preventing the reformation of biofilms.

Integration of advanced molecular diagnostic methods into clinical practice, although still limited, also offers a promising avenue for tailoring interventions based on individual patient needs and the progress of the healing. In particular, results gained by using advanced diagnostics tools, including DNA sequencing and confocal laser scanning microscopy, show a significant enhancement of the precision of biofilm detection and management, thus improving the overall treatment outcomes [28].

In light of these findings, chronic wound biofilm management clearly requires a multifactorial approach that incorporates aggressive debridement, targeted treatment, and cutting-edge diagnostic tools. As the chronic wound treatment field continues to grow, the ongoing adaptation of clinical practices and guidelines is likely to lead to further enhancement in the effectiveness of management strategies. New insights for healthcare providers and researchers are crucial for optimizing the management of chronic and acute wounds complicated by biofilms.

## 9. Conclusions

Biofilm presence in a chronic wound is considered a barrier to effective wound healing, as it prolongs inflammation, impedes tissue regeneration, and reduces the efficacy of conventional treatment. Research consistently shows that successful chronic wound management necessitates more than the simple application of antimicrobial agents; rather, it calls for a holistic approach that includes early biofilm detection, physical biofilm disruption, and targeted therapies. Advancements in molecular diagnosis, imaging-guided therapies, and innovative dressings have demonstrated promise; however, their application in clinical settings is restrained by accessibility and cost issues. Combined therapies that incorporate biofilm debridement through the utilization of biofilm-disrupting agents or NPWT are considered by far the most effective of the currently available strategies. The integration of novel technologies that target biofilms, such as electroceutical dressings and enzyme cocktails, could revolutionize chronic wound management. Incorporating these advancements into daily clinical practices could help in chronic wound management and reduce their adverse impacts on healthcare systems. In summary, this review offers an integrated view connecting microbial biofilm mechanisms with diagnostic and therapeutic innovations, aiming to highlight translational aspects where improved standardization and scalability could enhance clinical application.

## Figures and Tables

**Table 1:** Anti-biofilm therapeutics. Summary of biofilm-targeted therapeutic options for chronic wounds with reported outcomes, key cautions, and points of integration.

	Anti-Biofilm Mechanism	Reported Observation	Cautions	Integration	Ref.
Debridement	Physically disrupts biofilms and affect EPS	Structured repeated debridement linked to improved closure, enables antibiotics to penetrate	Requires expertise, pain and bleeding risks	Immediate topical antiseptic and dressing, post procedure	[56,58]
Topical antiseptics (iodine, polyhexanide, silver)	Bactericidal (broad spectrum), prevents biofilm reformation	Improved management of infection risk	Local irritation, products variability	After debridement	[10,63]
Advanced dressings (e.g., AQUACEL Ag^+^)	Prolonged antimicrobial activity, sequestration of exudate	Higher wound closure rates	Cost	As primary dressing post debridement	
					[57,64]
NPWT	Macro/micro-deformation, fluid exchange disrupts biofilm	Reduction in bioburden and exudate	Device availability, Reduce biofilm thickness and mass. Needs antibiotics to eliminate viable bacteria.	Between dressing change	[59,60,61]
Enzyme-based(e.g., glycoside hydrolases, DNase, dispersin B).	Targeted biofilm degradation	Improved bacterial killing and susceptibility	Stability formulation	After debridement, before topical antiseptic and systemic antimicrobial.	[65,66]
Electroceutical/E-stim	modify bacterial gene expression	Accelerate wound closure	Device access, protocol differences		[67]
				Adjunct to standard care	

## Data Availability

No new data were created in this review.

## Abbreviations

The following abbreviations are used in this manuscript:

ECM	Extracellular matrix
EPS	Extracellular polymeric substance
